# Analysis of Various Types of Glomerulonephritis with Crescents at a Single Center

**DOI:** 10.1155/2022/1749548

**Published:** 2022-05-09

**Authors:** Tomo Nakakita, Kenichi Akiyama, Kazunori Karasawa, Yoei Miyabe, Takahito Moriyama, Keiko Uchida, Kosaku Nitta

**Affiliations:** Department of Nephrology, Tokyo Women's Medical University, Tokyo, Japan

## Abstract

**Background:**

The importance of crescent formation in glomerulonephritis has increased. However, detailed analysis of crescentic glomerulonephritis in Asia is scarce. In addition, advances in serological diagnostic techniques (antineutrophil cytoplasmic and antiglomerular basement membrane autoantibodies) and early diagnosis have reduced the number of cases meeting the strict definition of crescentic glomerulonephritis (>50% of glomeruli are crescentic). Therefore, we analyzed the clinicopathological features and renal prognosis of glomerulonephritis cases that exhibited at least one crescentic lesion.

**Methods:**

We retrospectively evaluated 265 adult patients diagnosed with glomerulonephritis with at least one crescent formation based on the results of renal biopsy. We divided the patients into two groups based on the four types of glomerulonephritis, namely, the immune-complex (type II: IgA nephropathy, IgA vasculitis with nephritis, and lupus nephritis) and pauci-immune (type III: microscopic polyangiitis) groups. Factors affecting renal prognosis (end-stage renal failure requiring renal replacement therapy) were examined in a multivariate analysis using the Cox proportional hazards model. Kaplan–Meier curves and log-rank test were used to analyze and compare time from entry to renal death.

**Results:**

Renal prognosis differed significantly between the immune-complex and pauci-immune groups. Among the four types of glomerulonephritis, IgA nephropathy was the most prevalent. Multivariate analysis showed that renal function at renal biopsy and the ratio of global sclerosis independently predicted renal prognosis, but the type of glomerulonephritis was not a factor.

**Conclusions:**

Renal dysfunction at renal biopsy and the ratio of global sclerosis predicted renal prognosis, because it reflects the degree of irreversible renal damage. We also suspect that the formation of at least one crescentic lesion led to the development of these predictive factors, regardless of the type of glomerular disease and degree of crescent formation.

## 1. Introduction

Histopathologically, crescentic glomerulonephritis (CreGN) with cellular, fibrocellular, and fibrous crescent formation is typically observed in patients with rapidly progressive glomerulonephritis (RPGN) [1]. CreGN is defined as nephritis in which more than 50% of the glomeruli sampled in a renal biopsy show crescents [2]. Until recently, many reports have classified CreGN and/or RPGN into the following three types based on immunofluorescence microscopy findings: type I or antiglomerular basement membrane (GBM) disease was defined as a linear deposition of immunoglobulins along the GBM and is the typical presentation; type II, which included immunoglobulin A nephropathy (IgA-N) and lupus nephritis (LN), was defined as glomerular immune-complex deposition; and type III, or antineutrophil cytoplasmic autoantibody (ANCA)-related nephritis, which includes microscopic polyangiitis (MPA), was defined as a pauci-immune deposition [3, 4].

Recent improvements in diagnostic techniques, such as in the measurement method of serum ANCA titers, have enabled diagnosis before the exact pathological definition of CreGN is fulfilled [1]. Thus, in many cases, a diagnosis is made before renal biopsy results are obtained, even though the criteria do not meet the strict definition. This has led to an increasing number of RPGN cases over the past decade; however, it has been speculated that the number of CreGN cases fulfilling the strict criteria may not have increased [5]. Therefore, the number of cases with glomerulonephritis and crescent formation is expected to have increased, yet few reports have analyzed only cases of glomerulonephritis in which crescents were formed. Therefore, there is insufficient evidence to support that the present definition of CreGN accurately reflects the actual clinical situation.

The detailed molecular mechanism involved in crescent formation has not yet been elucidated. However, pathological findings commonly show glomerular loop ruptures, regardless of the underlying disease [6]. The presence and ratio of crescent formation in various glomerulonephritis types have recently been evaluated, and the ratio of crescent formation was added to the Oxford pathological classification of IgA-N [7]. The International Society of Nephrology and the Society of Renal Pathology classification defines crescents as active lesions in cases of LN [8]. Accordingly, we hypothesized that the presence of even one crescentic lesion could be an important factor in predicting renal prognosis and determining treatment for any type of underlying glomerulonephritis. Therefore, we conducted the present retrospective study of glomerulonephritis cases in which at least one crescent was detected using renal biopsy.

## 2. Materials and Methods

### 2.1. Patients

We examined cases at our hospital using the classification described in a previous report that classified and reported three types of CreGN [3, 4]. None of the cases of anti-GBM disease corresponded to the criteria for type I glomerulonephritis with crescents in our hospital among the three classifications. Patients who were serologically positive for anti-GBM antibodies but did not undergo renal biopsy were excluded. Type II glomerulonephritis with crescents was heterogeneous. According to this definition, IgA-N was the type observed most often, followed by LN and IgA vasculitis with nephritis (IgA-VN), accounting for 95% of cases with these three diseases. Other cases with type III glomerulonephritis with crescents were excluded because there were very few cases. In this study, we further analyzed type II glomerulonephritis with crescents by dividing it into three diseases (IgA-N, LN, and IgA-VN). Type III glomerulonephritis with crescents has regional characteristics. In Japan, it is predominantly MPA, and almost no other cases of vasculitis can be regarded as a single disease. Therefore, glomerulonephritis with crescents in our hospital was reclassified into four groups based on the presence of IgA-N, IgA-VN, LN, MPA, and the underlying disease.

Renal biopsy-proven MPA, IgA-N, IgA-VN, and LN with the observation of at least one crescent on histopathology results between January 2000 and April 2018 at the Tokyo Women's Medical University Hospital were included in this study. The findings of crescent formation included cellular, fibrocellular, and fibrous crescents. Renal biopsy was performed between April 2003 and April 2007. The exclusion criteria included patients aged under 16 years at the time of renal biopsy, patients with a follow-up period of less than one year, and patients with IgA-N who underwent tonsillectomy. The endpoints were dialysis induction, death, and one-year survival rate. A total of 196 cases were extracted, including 47 (24.0%) cases of MPA, 78 (39.8%) cases of IgA-N, 31 (15.8%) cases of IgA-VN, and 40 (20.4%) cases of LN. This study complied with the guidelines of the Declaration of Helsinki and was approved by our institutional ethics committee (**#4811**). We received verbal consent from all patients, and we provided them with the opportunity to opt out. This method of obtaining consent was approved by the ethics committee of Tokyo Women's Medical University.

### 2.2. Clinical Findings

Clinical data included sex, age, body mass index (BMI), systolic, diastolic, and mean arterial pressure (MAP), interval between renal biopsy onset (interval from onset), and laboratory data such as serum total protein, serum albumin (Alb), blood urea nitrogen, and serum creatinine levels; estimated glomerular filtration rate (eGFR); serum uric acid levels; total cholesterol, low-density lipoprotein-cholesterol, and triglyceride levels; IgG; IgA; IgM; C3; C4; CH50; urinary protein excretion; urinary red blood cell count (U-RBC); and complications (e.g., hypertension, dyslipidemia, and hyperuricemia). U-RBC was assessed using semi-quantitative analysis as a null count of RBC/high-power field (HPF) as follows: <1 RBC/20 HPF, 1 RBC/10–19 HPF, 1 RBC/5–9 HPF, 1 RBC/1–5 HPF, 1–5 RBCs/HPF, 5–9 RBCs/HPF, 10–19 RBCs/HPF, 20–29 RBCs/HPF, 30–49 RBCs/HPF, 50–99 RBCs/HPF, and ≥100 RBCs/HPF, and we selected the lowest number of RBCs in each grade as the U-RBC value. We also determined whether angiotensin-converting enzyme inhibitors, angiotensin II receptor blockers, or antiplatelet drugs were administered.

### 2.3. Histological Findings

Light microscopy (LM) findings were reviewed by expert renal pathologists at the time of the original report. The recorded LM findings included the following: global glomerulosclerosis percentage, crescent type (cellular/fibrocellular/fibrous), relative to the total number of glomeruli, and tubular atrophy and interstitial fibrosis based on the tubulointerstitial compartment. These findings were graded as follows: the extent of global glomerulosclerosis was graded into five categories: grade 0, lesion < 10%; grade 1, 10% ≤ lesion < 25%; grade 2, 25% ≤ lesion < 50%; grade 3, 50% ≤ lesion < 75%; and grade 4, lesion ≥ 75%. The extent of crescents was graded into five categories: grade 0, lesion < 10%; grade 1, 10% ≤ lesion < 25%; grade 2, 25%≤ lesion < 50%; grade 3, 50% ≤ lesion < 75%; and grade 4, lesion ≥ 75%. The extent of tubular atrophy and interstitial fibrosis was graded into four categories: grade 0, lesion < 5%; grade 1, 5% ≤ lesion < 20%; grade 2, 20% ≤ lesion < 50%; and grade 3, lesion ≥ 50%. The percentage of normal glomeruli was calculated as total glomeruli—global or segmental sclerotic lesions—crescents. The arteriosclerosis and arteriolosclerosis score was categorized as follows: none or low, 0; middle, 1; high, 2; and very high, 3. All specimens were obtained using percutaneous needle biopsy. Specimens were fixed with 10% phosphate-buffered formalin (pH 7.2), embedded in paraffin, and cut into 4-*μ*m-thick sections. The sections were stained with hematoxylin and eosin, periodic acid-Schiff, silver methenamine, and Masson trichrome for LM. Note that the average number of glomeruli collected in renal biopsies was 20 (3–38) for LN, 20 (6–53) for IgA-N, 23 (11–44) for IgA-VN, and 24 (8–63) for MPA. The percentage of crescent formation in each of the four glomerulonephritis was defined as C1 for less than 25%, C2 for greater than 25% and less than 50%, and C3 for greater than 50% (strict CreGN definition). Among 40 (%) LNs, 25 (62.5) had C1, 11 (27.5) had C2, and 4 (10.0) had C3. Of the 78 IgA-N cases, 58 (74.4) had C1, 17 (21.8) had C2, and 3 (3.8) had C3. Of the 31 IgA-VN cases, 27 (87.0) had C1, 2 (6.5) had C2, and 2 (6.5) had C3. Of the 44 MPA cases, 17 (38.6) had C1, 16 (36.4) had C2, and 11 (25.0) had C3. Three MPA cases had missing data, so the percentage of crescent formation was unknown.

### 2.4. Statistical Analyses

The two groups (type II and type III) and the four types of glomerulonephritis with crescents (MPA, IgA-N, IgA-VN, and LN) were compared. Each item was graded, and the factors contributing to renal prognosis (end-stage renal failure requiring renal replacement therapy) were analyzed using the Cox proportional hazards model. Subsequently, multivariate analysis was performed using factors that differed significantly in univariate analysis (*p* < 0.01). Data are expressed as mean ± standard deviation for normally distributed data and median ± interquartile range for non-normally distributed data and were analyzed using JMP® 14 software (SAS Institute Inc., Cary, NC, USA). The unpaired Student's *t*-test and Mann–Whitney *U* test were used to compare the clinical findings for normally and non-normally distributed data, respectively, whereas the chi-squared test was used to compare the clinical and histological grades and sex distributions at the time of renal biopsy.

The cumulative renal survival rate until the endpoint was calculated according to the Kaplan–Meier method and log-rank test. Statistical significance was set at *p* < 0.05.

## 3. Results

Almost all clinical findings in the 196 cases of IgA-N, IgA-VN, LN, and MPA differed significantly, except for MAP, BMI, and U-RBC. For IgA-N, the degree of U-RBC did not differ significantly, but the urinary protein excretion was lower and Alb level was preserved compared to the same values in the other three types of disease. LN was also more common in women, whereas IgA-VN was more common in men. Compared to the other three types of disease, MPA patients were older and had significantly more deteriorated renal function at biopsy. Approximately 80% of patients with IgA-N and IgA-VN were administered antiplatelet drugs, and 60% of patients with IgA-N were treated with angiotensin receptor blockers or angiotensin-converting enzyme inhibitors. Moreover, although some complications occurred, there was a significant difference only in those with diabetes mellitus. Furthermore, MPA had a significantly higher ratio of crescent formation, global sclerosis, and interstitial fibrosis (Table 1).

In a comparison of characteristics at renal biopsy between the two groups, type III patients were found to be significantly older than type II patients, have a lower proportion of men, and have renal impairment. Type II patients had significantly lower levels of complement factors and a significantly higher incidence of hypertension; moreover, several patients were treated with antiplatelet drugs. Renal pathological findings showed that type III patients exhibited a significantly higher ratio of crescent formation, glomerular sclerosis, and tubulointerstitial fibrosis (Table 2).

**Figure 1:**
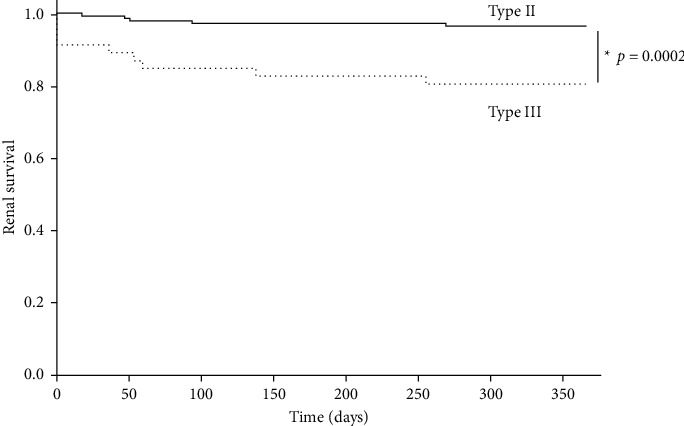
One-year renal survival in patients with type II and type III glomerulonephritis and crescents. Analysis of the 1-year survival rate using the Kaplan–Meier method shows that the renal prognosis of patients with type III glomerulonephritis and crescents was significantly worse (*p*=0.0002). The solid line indicates the type II glomerulonephritis with the crescent group, whereas the dotted line indicates the type III glomerulonephritis with the crescent group.

One-year renal survival rates for patients with type III glomerulonephritis with crescents were significantly worse than those for patients with type II glomerulonephritis and crescentic lesions (*p*=0.0002) (Figure 1). Univariate analysis revealed significant differences between the two groups in terms of disease, age, MAP, eGFR, total cholesterol, global sclerosis ratio, tubular atrophy, interstitial fibrosis, and the ratio of normal glomeruli. The proportion of crescents tended to be higher in type III patients but did not differ significantly. In multivariate analysis with renal prognosis as the outcome, only renal function, total cholesterol level, and global sclerotic lesions differed significantly (Table 3).

**Figure 2:**
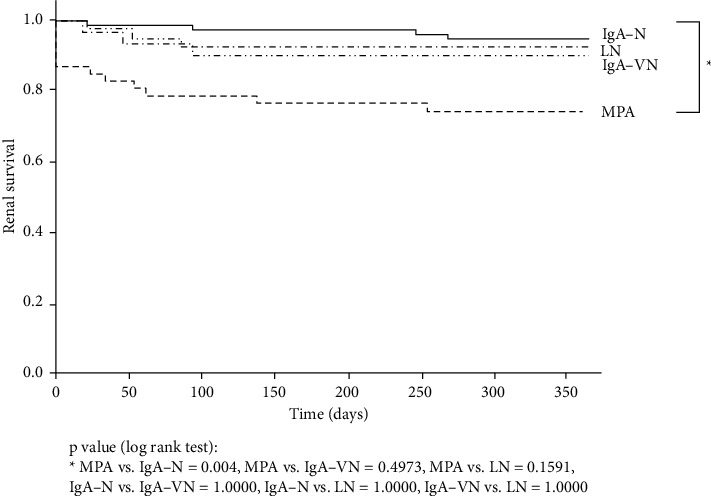
One-year renal survival according to the four classifications of glomerulonephritis with crescents. One-year renal survival was analyzed using the Kaplan–Meier method, and a significant difference was only observed between MPA and IgA-N (*p*=0.004). The solid line indicates IgA-N. The dotted lines indicate type III MPA. MPA, microscopic polyangiitis; IgA-N, immunoglobulin A nephropathy; LN, lupus nephritis; IgA-VN, IgA vasculitis with nephritis.

Only IgA-N and MPA showed a significant difference when renal prognosis was compared between the four disease types (*p*=0.004) (Figure 2). Univariate analysis of IgA-N and MPA revealed significant differences in underlying disease, age, MAP, eGFR, total cholesterol, global sclerosis ratio, tubular atrophy, interstitial fibrosis, and the ratio of normal glomeruli. Underlying disease, renal function, global sclerotic lesion percentage, tubular atrophy and interstitial lesions, and percentage of normal glomeruli remained factors in the multivariate analysis with renal prognosis as the outcome, and only eGFR and global sclerotic lesions were significant factors (Table 4).

## 4. Discussion

The injury mechanism causing glomerular loop rupture remains the same regardless of the underlying disease triggering crescent formation. The presence or absence of crescent formation is thought to affect renal prognosis, which has led to the recent recognition of crescent formation as an independent renal function exacerbation factor for each glomerular disease [6, 9].

Recent reports of CreGN cases fulfilling the strict definition suggest that type I CreGN has a significantly worse renal prognosis compared with type II and type III CreGN [3, 4]. This may be due to differences in the underlying disease that triggers crescent formation; however, to our knowledge, no reports have outlined the detailed mechanisms to date.

In this study, only 12 and 14 cases of type II glomerulonephritis with crescents and type III glomerulonephritis with crescents, respectively, fulfilled the strict definition of CreGN, whereas type II glomerulonephritis with crescents did not include postinfectious glomerulonephritis or RPGN-type IgA-N. Therefore, unlike the previous report on CreGN, this was considered a unique finding in cases of glomerulonephritis with crescents [2–4]. However, even in the present study, type III glomerulonephritis with crescents had a significantly poorer renal prognosis compared to type II glomerulonephritis with crescents. Our findings strongly suggest that the difference between the underlying type II and type III glomerulonephritis that triggers crescent formation may significantly affect renal prognosis.

Surprisingly, multivariate analysis did not identify the type of underlying disease as an exacerbating factor for renal prognosis when type II glomerulonephritis and type III glomerulonephritis with crescents were compared. In all multivariate analyses, eGFR calculated using creatinine levels at the time of renal biopsy and global sclerosis on renal biopsy were extracted as factors exacerbating renal prognosis. However, it is worth noting that this study did not identify any factors that are clinically suggested to exacerbate renal prognosis, including age, blood pressure, or proteinuria [10, 11]. Histologically, it was also contrary to our expectation that the crescent ratio, tubular atrophy, interstitial fibrosis, and atherosclerotic lesions were not identified as renal prognosis exacerbation factors [12]. These results suggest that the presence or absence of global sclerosis is strongly correlated with renal prognosis in cases of glomerulonephritis with crescents. In our study, IgA-N was the most common case. For IgA-N, the Oxford classification (MEST) was proposed in 2009 as a criterion to accurately predict renal prognosis from pathological diagnosis [13]. Subsequently, the importance of crescents formation (especially crescents >25% of glomeruli, i.e., C2) was recognized and the MEST-C score was proposed [14]. For IgA-VN, a pediatric case was reported in which renal prognosis was examined using MEST-C scoring, and S1 and T1 and T2 were significantly associated with renal prognosis, while C2 lesions were associated with decreased renal function at the time of renal biopsy [15]. In our study, the degree of crescent formation was not significantly associated with renal prognosis on multivariate analysis. This may be due to the fact that we did not include cases without crescent formation, that is, C0 on the MEST-C score, and that all cases were treated with immunosuppressive drugs, including methylprednisolone pulse therapy [16]. Of the present cases, 15 of 40 LN (37.5%), 20 of 78 IgA-N (25.6%), 4 of 31 IgA-VNs (12.9%), and 27 of 44 MPA (51.4%) had crescents formation >25%. Three cases of MPA could not be evaluated due to missing data.

Several reports have been written on the relationship between global sclerosis and the ratio of crescent formation in ANCA-associated vasculitis-associated glomerulonephritis. The sclerotic type of vasculitis-associated glomerulonephritis consisting of primarily global sclerosis reportedly had a worse renal prognosis than the crescentic type with a large crescentic ratio, similar to the results of the present study [17, 18]. The percentage of normal glomeruli as well as global glomerulosclerosis was also considered important in previous reports [17, 19]; however, multivariate analysis of the percentage of normal glomeruli in patients with MPA only revealed that total nodal sclerosis was a significant factor associated with renal prognosis (Supplementary Table 1). The reason that the ratio of crescent formation does not directly correlate with renal prognosis can be understood as a reflection of the difference in response to the treatment of cellular crescent and global sclerosis.

However, the final stage of the crescent glomerular injury process is a compression of the glomerulus from the outside, resulting in glomerular collapse, which rapidly induces global sclerosis, similar to a collapsing variant in focal global sclerosis [20, 21]. In previous reports of CreGN, type I and type III CreGN were aggressive to the extent that even patients with little or no crescent formation were suggested to be considered for substantial immunosuppressive treatment [22]. Therefore, clinicians should provide aggressive immunosuppressive treatment as soon as possible in cases of glomerulonephritis when a renal biopsy reveals even one crescent lesion.

Our study had several limitations. First, the observation period for studying renal prognosis was very short. There were no cases of type I CreGN, ANCA-associated vasculitis is extremely similar to MPA in Japan, unlike in Europe and the United States, and the treatment regimen is mild (Supplementary Table 2). Despite these limitations, the renal function (eGFR) at the time of renal biopsy and global glomerulosclerosis were significant predictors of renal prognosis in crescentic glomerulonephritis in our analysis, as in previous studies [3, 18, 19].

## 5. Conclusions

In cases of glomerulonephritis with at least one crescentic lesion, global sclerosis and eGFR calculated using the creatinine value at renal biopsy were independent factors for exacerbated renal prognosis, regardless of the underlying disease.

## Figures and Tables

**Table 1:** Clinical and pathological findings according to the four classifications of rapid progressive glomerulonephritis.

	Type II (*n* = 149)			Type III (*n* = 47)	*p* value
	LN (40)	IgA–N (78)	IgA–VN (31)	MPA (47)	
Clinical findings					
Age (years)	39.5 (32.3–50)	33.5 (26–42.3)	30.5 (24.5–44)	64 (54–70.5)	<0.0001
Sex (male/female)	7/33 (M:18%)	37/41 (M:47%)	22/9 (M:71%)	18/30 (M:38%)	0.0002
Mean arterial pressure (mmHg)	91 (84.3–107.2)	92.2 (84.9–100)	93 (81.7–98.7)	96 (82.7–105.8)	0.6150
Body mass index	22.0 (20.5–23.6)	21.3 (19.7–23.4)	21.4 (19.3–24.4)	21.6 (19.9–24.3)	0.5850
Total serum protein (g/dL)	5.8 (4.7–6.2)	6.6 (6.1–7)	6.5 (5.9–6.9)	6.6 (6.2–7.2)	<0.0001
Serum albumin (g/dL)	2.9 (2.2–3.5)	3.9 (3.5–4.2)	3.7 (3.2–4.2)	3.2 (2.6–4)	<0.0001
Blood urea nitrogen (mg/dL)	18 (13.4–23.9)	14 (12.1–17.8)	13.1 (11.9–17.2)	26.7 (18.2–43.2)	<0.0001
Serum creatinine (mg/dL)	0.79 (0.62–1.09)	0.78 (0.67–1.04)	0.78 (0.67–0.91)	1.71 (1.03–2.76)	<0.0001
Estimated GFR (mL/min/1.73 m^2^)	71.4 (46.3–93.1)	75.9 (57–95.6)	85.5 (69.5–107.8)	25 (16.8–42.9)	<0.0001
Urine protein (g/g creatinine)	1.9 (0.88–4.7)	1.19 (0.66–2.34)	2 (0.78–3.37)	1.54 (0.74–2.75)	0.0340
Urine red blood cell count (counts/HPF)	20 (5–30)	20 (10–42.5)	30 (10–50)	20 (10–30)	0.2950
IgG (mg/dL)	1183 (849–1685)	1049 (850–1298)	893.5 (737–1110)	1338 (1047–1626)	0.0002
IgA (mg/dL)	232 (170.5–305)	283 (221–386)	251 (196.3–369.5)	215 (166–281)	0.0027
IgM (mg/dL)	73.5 (48–101)	128 (88–168)	104.5 (84–153.3)	89 (58–115)	<0.0001
CH50 (mg/dL)	26.9 (20–36.3)	40 (35.2–47.6)	47.5 (44.1–53)	51.3 (43.6–59.0)	<0.0001
C3 (mg/dL)	50.1 (36–72.3)	94.6 (83.9–108.6)	110 (91.4–121)	106.6 (95.8–121)	<0.0001
C4 (mg/dL)	9.45 (3.9–17.2)	22.3 (19.7–27.6)	23.6 (20–29.9)	26.9 (22.0–34.1)	<0.0001
Complications (%)					
Diabetes mellitus	3 (7.5)	2 (2.6)	4 (12.9)	5 (10.4)	0.0786
Hypertension	10 (25)	22 (28.2)	8 (25.8)	20 (41.7)	0.0698
Dyslipidemia	11 (27.5)	20 (25.6)	8 (25.8)	24 (50)	0.8289
Hyperuricemia	5 (12.5)	9 (11.5)	1 (3.2)	9 (18.8)	0.2362
Treatment (%)					
Antiplatelet drug	21 (52.5)	62 (79.5)	24 (77.4)	16 (33.3)	<0.0001
ACEI/ARB	13 (32.5)	47 (60.3)	14 (45.2)	25 (52.1)	0.0282
Pathological finding					
Percentage of global glomerulosclerosis (%)	5.1 (0–16.3)	11.1 (2.6–25.4)	8.3 (0–22.2)	27.8 (6.7–38.4)	<0.0001
Percentage of crescents (Cellular/fibrocellular/fibrous) (%)	15.1 (7.7–36.7)	16.7 (10.2–26.5)	14.3 (9.1–18.2)	28 (20.6–51.7)	<0.0001
Tubulointerstitial lesions^a^ (0/1/2/3)	14/18/4/2	11/39/25/3	9/20/3/0	9/11/11/12	0.0001
Arteriosclerosis^b^ (0/1/2/3)	25/7/6/2	40/17/14/1	20/5/4/2	16/13/11/4	0.0724
Arteriolar sclerosis^b^ (0/1/2/3)	27/9/4/0	33/26/16/2	18/8/4/1	16/19/8/1	0.0283
Percentage of normal glomeruli (%)	69.0 (55.5–86.0)	64.0 (44.8–75.0)	71.0 (60.0–83.0)	34.5 (21.0–54.5)	<0.0001

GFR, Glomerular filtration rate, HPF, high-power field; ACEI, angiotensin-converting enzyme inhibitor; ARB, angiotensin II receptor blocker. ^a^Tubular atrophy and interstitial fibrosis score: <5%, 0; 5 ≤ lesion <20, 1; 20 ≤ lesion < 50, 2; ≥50, 3. ^b^Arteriosclerosis and arteriolosclerosis score: none or low, 0; middle, 1; high, 2; and very high 3.

**Table 2:** Clinical and pathological findings in type II and type III rapid progressive glomerulonephritis at the time of renal biopsy.

	Type II (*n* = 149)	Type III (*n* = 47)	*p* value
Clinical findings			
Age (years)	34 (26–45)	64 (54–70.5)	<0.0001
Sex (male/female)	66/83 (M: 44%)	18/30 (M: 38%)	<0.0001
Mean arterial pressure (mmHg)	92.3 (84.7–100)	96 (82.7–105.8)	0.2065
Body mass index	21.5 (19.8–23.5)	21.6 (19.9–24.3)	0.6903
Total serum protein (g/dL)	6.3 (5.7–6.9)	6.6 (6.2–7.2)	0.0238
Serum albumin (g/dL)	3.7 (3.1–4.1)	3.2 (2.6–4)	0.0438
Blood urea nitrogen (mg/dL)	14.5 (12.1–19.4)	26.7 (18.2–43.2)	<0.0001
Serum creatinine (mg/dL)	0.79 (0.67–1.02)	1.71 (1.03–2.76)	<0.0001
Estimated GFR (mL/min/1.73 m^2^)	75.8 (59.1–98.0)	25 (16.8–42.9)	<0.0001
Urine protein (g/g creatinine)	1.33 (0.71–2.73)	1.54 (0.74–2.75)	0.7207
Urine red blood cell count (counts/HPF)	20 (10–50)	20 (10–30)	0.5211
IgG (mg/dL)	1037 (807–1323)	1338 (1047–1626)	0.0006
IgA (mg/dL)	267 (198–366)	215 (166–281)	0.0037
IgM (mg/dL)	106 (79–149)	89 (58–115)	0.0269
CH50 (mg/dL)	39.5 (32.1–47.8)	51.3 (43.6–59.0)	<0.0001
C3 (mg/dL)	91.5 (72.9–109.5)	106.6 (95.8–121)	<0.0001
C4 (mg/dL)	21.5 (15.3–26.9)	26.9 (22.0–34.1)	<0.0001
Complications (%)			
Diabetes mellitus	9 (6)	5 (10.4)	0.2912
Hypertension	40 (26.8)	20 (41.7)	0.0086
Dyslipidemia	39 (26.2)	24 (50)	0.5206
Hyperuricemia	15 (10.1)	9 (18.8)	0.0422
Treatment (%)			
Antiplatelet drug	107 (72)	16 (33.3)	0.0002
ACEI/ARB	74 (50)	25 (52.1)	0.3300
Pathological findings			
Percentage of global glomerulosclerosis (%)	10.0 (0–22.2)	27.8 (6.7–38.4)	<0.0001
Percentage of crescents (Cellular/fibrocellular/fibrous) (%)	15.9 (9.1–27.3)	28 (20.6–51.7)	<0.0001
Tubulointerstitial lesions^a^ (0/1/2/3)	34/7732/5	9/11/11/12	0.0001
Arteriosclerosis^b^ (0/1/2/3)	85/2924/5	16/13/11/4	0.0094
Arteriolar sclerosis^b^ (0/1/2/3)	78/43/24/3	16/19/8/1	0.1548
Percentage of normal glomeruli (%)	67.0 (49.0–80.5)	34.5 (21.0–54.5)	<0.0001

GFR, glomerular filtration rate, HPF, high-power field; ACEI, angiotensin-converting enzyme inhibitor; ARB, angiotensin II receptor blocker. ^a^Tubular atrophy and interstitial fibrosis score: <5%, 0; 5 ≤ lesion < 20, 1; 20 ≤ lesion < 50, 2;  ≥ 50, 3. ^b^Arteriosclerosis and arteriolosclerosis score: none or low, 0; middle, 1; high, 2; and very high, 3.

**Table 3:** Univariate and multivariate analysis of factors affecting renal prognosis between type II and type III rapid progressive glomerulonephritis.

	Univariate analysis			Multivariate analysis		
	HR	**95**% CI	*p* value	HR	**95**% CI	*p* value
Type III (vs. type II)	15.08	3.08–73.9	0.0001	0.07	0.01–2.52	0.10522
Age (15 years of increase)	2.60	1.45–4.67	0.0005	0.67	0.15–2.99	0.59377
Male (vs. Female)	0.92	0.25–3.38	0.9036			
MAP (10 mm Hg increase)	2.05	1.15–3.67	0.0125			
BMI (3 kg/m2 increase)	1.23	0.70–2.16	0.463			
eGFR (30 mL/min/1.73 m2 decrease)	17.57	2.55–121	<0.0001	8.57 E+08	–	0.00012
T-chol (30 mg/dL increase)	0.46	0.26–0.81	0.0034	0.29	0.10–0.86	0.00448
TG (30 mg/dL increase)	0.35	0.07–1.82	0.0541			
U-Prot (0.5 g/g Cr increase)	1.13	0.88–1.45	0.3329			
U-RBC (25 counts/HPF increase)	0.98	0.54–1.76	0.9348			
ACEI/ARB use (vs. nonuse)	0.89	0.22–3.65	0.8677			
Antiplatelet drugs use (vs. nonuse)	0.75	0.16–3.46	0.7168			
Percentage of global glomerulosclerosis^a^	1.11	1.06–1.16	<0.0001	8.99	1.40–57.9	0.00176
Percentage of crescents^b^ (cellular/fibrocellular/fibrous)	1.83	1.01–3.33	0.0486			
Tubular atrophy and interstitial fibrosis^c^	6.69	2.22–22.2	0.0001	0.83	0.19–3.55	0.80201
Percentage of normal glomeruli^d^	0.22	0.08–0.58	0.0004	1.84	0.28–12.2	0.51339
Arteriosclerosis score^e^	1.83	1.83–0.95	0.071			
Arteriolosclerosis score^e^	2.67	1.26–6.03	0.0111			

HR, hazard ratio; CI, confidence interval; MAP, mean arterial pressure; BMI, body mass index; Alb, serum albumin; eGFR, estimated glomerular filtration rate; T-chol, total cholesterol; TG, triglycerides; U-Prot, total urine protein; U-RBC, urine red blood cell count; ACEI, angiotensin-converting enzyme inhibitor; ARB, angiotensin II receptor blocker. ^a^Global glomerulosclerosis score: < 10%, 0; 10 ≤ lesion < 25%, 1; 25 ≤ lesion < 50%, 2; 50 ≤ lesion < 75%, 3; ≥75%, 4. ^b^Crescent score: <10%, 0; 10 ≤ lesion < 25%, 1; 25 ≤ lesion < 50%, 2; 50 ≤ lesion < 75%, 3; ≥75%, 4. ^c^Tubular atrophy and interstitial fibrosis score: <5%, 0; 5 ≤ lesion < 20, 1; 20 ≤ lesion < 50, 2; ≥50, 3. ^d^Normal glomeruli score: > 75%, 0; 50 < lesion ≤ 75, 1; 25 < lesion ≤ 50, 2; ≤ 25, 3. ^e^Arteriosclerosis and arteriolosclerosis score: none or low, 0; middle, 1; high, 2; and very high, 3.

**Table 4:** Univariate and multivariate analysis of factors affecting renal prognosis microscopic polyangiitis and immunoglobulin A nephropathy.

	Univariate analysis			Multivariate analysis		
	HR	**95**% CI	*p* value	HR	**95**% CI	*p* value
MPA (vs. IgA-N)	15.8	1.91–131	0.0009	0.35	0.01–37.9	0.65774
Age (15 years increase)	2.01	1.13–3.60	0.0129			
Male (vs. Female)	0.64	0.15–2.67	0.5302			
MAP (10 mm Hg increase)	2.06	1.08–3.93	0.0225			
BMI (3 kg/m2 increase)	1.10	0.61–1.99	0.7415			
eGFR (30 mL/min/1.73 m2 decrease)	13.0	1.88–90.5	<0.0001	4.E+0.7	–	0.04942
T-chol (30 mg/dL increase)	0.50	0.28–0.92	0.0170			
TG (30 mg/dL increase)	0.50	0.11–2.33	0.2424			
U-Prot (0.5 g/g Cr increase)	1.30	0.98–1.73	0.0907			
U-RBC (25 counts/HPF increase)	0.89	0.47–1.70	0.7247			
ACEI/ARB use (vs. nonuse)	0.88	0.19–4.13	0.8741			
Antiplatelet drugs use (vs. nonuse)	1.05	0.18–6.01	0.9526			
Percentage of global glomerulosclerosis^a^	74.3	6.89–803	<0.0001	23.3	1.41–386	0.00127
Percentage of crescents^b^ (cellular/fibrocellular/fibrous)	1.96	0.98–3.89	0.0564			
Tubular atrophy and interstitial fibrosis^c^	10.0	2.42–41.7	0.0001	2.51	0.50–12.6	0.23657
Percentage of normal glomeruli^d^	0.08	0.02–0.38	<0.0001	0.38	0.02–7.74	0.53297
Arteriosclerosis score^e^	1.89	0.90–4.10	0.0903			
Arteriolosclerosis score^e^	2.60	1.13–6.57	0.0252			

HR, hazard ratio; CI, confidence interval; MAP, mean arterial pressure; BMI, body mass index; Alb, serum albumin; eGFR, estimated glomerular filtration rate; T-chol, total cholesterol; TG, triglycerides; U-Prot, total urine protein; U-RBC, urine red blood cell count; ACEI, angiotensin-converting enzyme inhibitor; ARB, angiotensin II receptor blocker. ^a^Global glomerulosclerosis score: <10%, 0; 10 ≤ lesion < 25%, 1; 25 ≤ lesion < 50%, 2; 50 ≤ lesion < 75%, 3; ≥75%, 4. ^b^Crescent score: <10%, 0; 10 ≤ lesion < 25%, 1; 25 ≤ lesion < 50%, 2; 50 ≤ lesion < 75%, 3; ≥75%, 4. ^c^Tubular atrophy and interstitial fibrosis score: <5%, 0; 5 ≤ lesion < 20, 1; 20 ≤ lesion < 50, 2; ≥50, 3. ^d^Normal glomeruli score: >75%, 0; 50 < lesion ≤ 75, 1; 25 < lesion ≤ 50, 2; ≤ 25, 3. ^e^Arteriosclerosis and arteriolosclerosis score: none or low, 0; middle, 1; high, 2; and very high 3.

## Data Availability

All data concerning each case are presented in the manuscript.

## Abbreviations

IgA: Immunoglobulin A
RPGN: Rapid progressive glomerulonephritis
CreGN: Crescentic glomerulonephritis
GBM: Glomerular basement membrane
IgA-N: Immunoglobulin A nephropathy
ANCA: Anti-neutrophil cytoplasmic autoantibody
MPA: Microscopic polyangiitis
Alb: Serum albumin
BMI: Body mass index
MAP: Mean arterial pressure;
eGFR: estimated glomerular filtration rate
U-RBC: Urinary red blood cell count.

## Supplementary Materials

Supplementary Materials Supplementary Table 1: Univariate and multivariate analysis of factors affecting renal prognosis microscopic polyangiitis. Supplementary Table 2: Clinical score, clinical grade, and treatment regimen for microscopic polyangiitis therapy in Japan.
